# Arecoline induces TNF-alpha production and Zonula Occludens-1 redistribution in mouse Sertoli TM4 cells

**DOI:** 10.1186/s12929-014-0093-z

**Published:** 2014-09-09

**Authors:** Tzer-Min Kuo, Shun-Yuan Luo, Shang-Lun Chiang, Chi-Pin Lee, Yu-Fan Liu, Jan-Gowth Chang, Ming-Hsui Tsai, Ying-Chin Ko

**Affiliations:** Environment-Omics-Disease Research Centre, China Medical University Hospital, Taichung, Taiwan; Graduate Institute of Clinical Medicine Science, China Medical University, Taichung, Taiwan; Department of Chemistry, National Chung Hsing University, Taichung, Taiwan; Graduate Institute of Medicine, College of Medicine, Kaohsiung Medical University, Kaohsiung, Taiwan; Department of BioMedical Sciences, Chung Shan Medical University, Taichung, Taiwan; Center of RNA Biology and Clinical Application, China Medical University Hospital, Taichung, Taiwan; Department of Otorhinolaryngology, China Medical University Hospital, Taichung, Taiwan

**Keywords:** Arecoline, Testis, Serotli cell, TNF-alpha, ZO-1, Micro-Western Array

## Abstract

**Background:**

Arecoline, a major alkaloid in Areca nut has the ability to induce oxidative stress. The effect of Areca nut, arecoline on reducing sperm quality and quantity were documented previously using several animal models. Junction disruption by down-regulation of the junction-adhesive protein via oxidative stress is an important route mediating abnormal spermatogenesis. Therefore, in this present study, we investigated the functional role of arecoline on junctional proteins.

**Results:**

To analyze direct effects of arecoline on testis cells, confluent mouse testicular Sertoli cell line TM4 was exposed to arecoline. Arecoline decreased insoluble zonula occludens-1 (ZO-1) protein expression in TM4 cells, however, arecoline treatment increased TNF-alpha production in both TM4 and monocytic THP1 cells. In addition, ERK1/2 inhibitor PD98059 reversed arecoline effects on TNF-alpha and ZO-1.

**Conclusions:**

Arecoline increases the production of TNF-alpha and induces protein redistribution of ZO-1. All these results explain the role of arecoline in male reproductive dysfunction, besides its cytotoxic induction.

**Electronic supplementary material:**

The online version of this article (doi:10.1186/s12929-014-0093-z) contains supplementary material, which is available to authorized users.

## Background

Betel quid chewing is prevalent among Asian populations and migrant Asian communities, with about 600 million users reported worldwide [1]. Along with tobacco or alcohol consumption, betel quid chewing ranks as a common addiction [2]. Areca nut in betel quid has been recognized as a Group I carcinogen to humans by the International Agency for Research on Cancer (IARC), World Health Organization [3]. Research on chemical constituents identified arecoline, arecaidine, guvacoline and guvacine as its chief alkaloids, arecoline as most abundant and active [4]. Numerous works demonstrated its mutagenicity, genotoxicity and cytotoxicity using various experimental models [5,6]. Oxidative stress has been proven to act as a central role in cell death, gene regulation, and inflammatory processes via arecoline [7,8].

Apart from this, epidemic and experimental studies have shown that betel quid chewing or areca nut contents displayed harmful effecs for reproductive function. We and others showed that, betel quid chewing during pregnancy had a significant effect on birth outcomes: e.g., sex ratio at birth, lower birth weight, reduced birth length [9,10]. Exposure of Swiss albino mice to betel quid displayed embryotoxicity [11]. In addition, arecoline exposure induced micronuclei and cytokinesis in Chinese hamster ovary cells [12]. Both areca nut and arecoline induced male reproductive toxicity by *in vitro* or *in vivo* animal models: e.g., inhibition of male sexual behavior, abnormal sperm head shape, reduced sperm count and motility [13-17]. All these findings support a hypothesis of areca nut’s toxic effect on human reproductive functions. Focusing on male reproductive function, we primarily concluded that such dysfunction via areca nut might emanate from reduction in quantity and quality of sperm, based on those observations. Nonetheless, a pivotal question arose about how areca nut affects sperm. While our study indicated that, areca nut administration generated reactive oxygen species (ROS)-related oxidative stress in rat testis [16], current evidence is still limited, thus meriting research on direct molecular mechanism(s) of areca nut or arecoline in male reproductive regulation.

In testis, blood-testis barrier (BTB) and seminiferous tubules, is an essential microenvironment for spermatogenesis [18]. Disruption of BTB junction integrity is one major issue in studying molecular mechanisms of male reproductive dysfunction via toxicants (Adjudin, Aspirin, Bisphenol A, Cadmium, etc.) [19]. Previous studies on their molecular mechanisms have indicated that oxidative stress is commonly induced in testis via phosphatidylinositol 3-kinase (PI3K) or mitogen-activated protein kinase (MAPK) signaling pathways [19,20]. These signaling pathway up-regulates c-Src kinase activity or production of pro-inflammatory cytokines (TNF-alpha, TGF-beta2, IL-6, etc.), which further distorts junction integrity by decreasing or redistributing junction proteins and subsequently damage sperm counts. Tight junctions between adjacent Sertoli cells and epididymal epithelia in testis are critical junction types in BTB formation. Zonula occludens (ZO-1), a member of the membrane-associated guanylate kinase (MAGUK) homologue protein family, is a tight junction protein [21]. ZO-1 has been reported as a target protein of several toxicants in BTB disruption [22,23].

This study investigated the molecular mechanism (s) by how arecoline adversely affects male reproduction. Using a mouse testis cell line TM4, effects of arecoline on reproductive gene expressions or signaling activation were examined. We further investigated the effect of arecoline on inducing TNF-alpha production and ZO-1 protein redistribution. Our study unearths clues for possible mechanisms of male reproductive dysfunction by areca nut or arecoline.

## Methods

### Cell culture and viability assay

TM4 (mouse testicular Sertoli) and THP1 (human monocytic leukemia) cells purchased from Bioresource Collection and Research Center (BCRC, Taipei, Taiwan) were maintained in DMEM and RPMI 1640 medium, supplemented with 10% fetal bovine serum at 37°C in a 5% CO2 incubator. For measurement of cell viability, CytoTox-ONE™ Homogeneous Membrane Integrity Assay (LDH activity) and CellTiter 96 Aqueous One Solution Cell Proliferation Assay (MTS Assay) were performed according to manufacturer’s protocol (Promega Corporation, Madison, WI).

### Reagents and antibodies

Arecoline hydrobromide purchased from Sigma-Aldrich (St. Louis, MO) used primary antibodies for Western blot: anti-Phospho-Erk1/2 (Thr202/Tyr204) (Millipore, Temecula, CA), anti-Erk1/2 (Cell Signaling Technology, Danvers, MA), anti-Phospho-JNK (Millipore Corp., Billerica, MA), anti-Phospho-IkappaB-alpha (abcam, Cambridge, UK), anti-PP2A (abcam, Cambridge, UK), anti-Phospho-STAT-1 (Millipore Corp., Billerica, MA), anti-ZO-1 (Invitrogen Corporation, Carlsbad, CA) and anti-GAPDH (GeneTex Inc., Hsinchu City, Taiwan). ERK1/2 MAPK inhibitor PD98059 was purchased from Calbiochem (San Diego, CA).

### Micro-Western Array analysis

Confluent TM4 cells were treated 400 μM arecoline for 10 or 60 minutes. Micro-Western arrays were performed to detect the protein phosphorylation or expression by the Micro-Western Array core facility of National Health Research Institutes (NHRI, Miaoli County, Taiwan). Used 48 antibodies are listed in Additional file 1: Table S1. The methods were described previously [24,25].

### Western blotting analysis

To detect phosphorylated proteins, cells lysates were prepared in lysis buffer A (50 mM Tris–HCl, pH 7.4, 100 mM NaCl, 1 mM EDTA, 10 mM magnesium acetate, 1% NP-40) containing a cocktail of protease inhibitors (Roche, Mannheim, Germany). Total protein (100 μg) was separated by SDS-polyacryamide gel and electrotransferred to polyvinylidene fluoride membranes (Millipore, Billerica, MA). Membranes were incubated with primary antibodies as indicated and peroxidase-conjugated secondary antibodies, protein signals detected by enhanced chemiluminescence reagent (Millipore, Billerica, MA). For detection of ZO-1, cells were lysed in lysis buffer B (50 mM Tris–HCl, pH 7.4, 100 mM NaCl, 1 mM EDTA, 10 mM magnesium acetate, 1.0% Triton X-100, protease inhibitors cocktail, and 1 mM PMSF) on ice for 10 minutes. Extracts were centrifuged at 15600 × g for 5 min at 4°C to sediment the insoluble fraction. The pellet was re-suspended in lysis buffer B and mixed with an equal volume of 4 × Laemmli’s sample buffer. Sample loading of insoluble fraction was normalized by protein concentration of soluble fraction.

### Reverse transcription-polymerase chain reaction and real-time quantitative polymerase chain reaction (RT-qPCR)

For determination of RNA expression, cells were harvested in the indicated time points and treated with TRIzol reagent (Invitrogen Corporation, Carlsbad, CA) for RNA extraction according to the manufacturer’s protocol. The cDNA templates were obtained by reverse transcription of total RNA with random primer and high-capacity cDNA reverse transcription kit (Applied Biosystems, CA). The products were then subjected to quantitative real-time RT-PCR analysis with the specific primer pairs performed by an ABI StepOnePlus™ Real-Time PCR Systems (Applied Biosystems, CA) using Power SYBR® Green PCR Master Mix (Invitrogen Corporation, MD). Primer sequences were: (1) mouse TNF-alpha forward primer: 5′-GACGTGGAAGTGGCAGAAGAG-3′, reverse primer: 5′-TGCCACAAGCAGGAATGAGA-3′ [26]. (2) human TNF-alpha forward primer: 5′-CAGCCTCTTCTCCTTCCTGAT-3′, reverse primer: 5′-GCCAGAGGGCTGATTAGAGA-3′ [27]. (3) mouse ZO-1 forward primer: 5′-GGAGCTACGCTTGCCACACT-3′, reverse primer: 5′-GGTCAATCAGGACAGAAACACAGT-3′ [28]. (4) mouse GAPDH forward primer: 5′-TCTCCCTCACAATTTCCATCCCAG-3′, reverse primer: 5′-GGGTGCAGCGAACTTTATTGATGG-3′ [26]. (5) human GAPDH forward primer: 5′-AGCCACATCGCTCAGACAC-3′, human GAPDH reverse primer: 5′-GCCCAATACGACCAAATCC-3′ [29].

### Determination of soluble mouse and human TNF-alpha

For measurement of soluble TNF-alpha in culture medium, confluent TM4 and THP1 (2 × 10^6^) cells were treated with indicated concentration of arecoline for 6 hours in six-well plates in 1.5 mL of culture medium per well. Levels of mouse and human TNF-alpha in culture medium were gauged by individual enzyme-linked immunosorbent assay (ELISA) according to manufacteurer’s protocol (Invitrogen Corporation, Frederick, MD). 50 μL (TM4 cells) and 100 μL (THP1 cells) of culture medium and was added to well for those ELISA assay. Sensitivity of mouse and human TNF assays were 3 and 1.7 pg/ml respectively. Detection ranges of two assays were 19.5-1250 (mouse) and 15.6-1000 (human) pg⁄ml. No species cross-reactivity was observed in both two assays.

### Immunofluorescence (IF) detection of ZO-1

To stain for ZO-1, treated cells were fixed in 3.75% formaldehyde for 10 min, then blocked with 1% BSA in PBS for 1 hour at room temperature. Slides were incubated with rabbit antibodies against ZO-1 (1:100 dilution) for 1 hour, followed by fluorescein-conjugated anti-rabbit antibody (Jackson ImmunoResearch Laboratories, Inc., West Grove, PA) (1:200 dilution) for 1 hour. Between incubations, samples were washed three times for 10 minutes with PBS, slides viewed by fluorescent microscopy (Axio Observer. A1, Zess).

## Results

### Arecoline induces redistribution of ZO-1 directly

**Figure 1:**
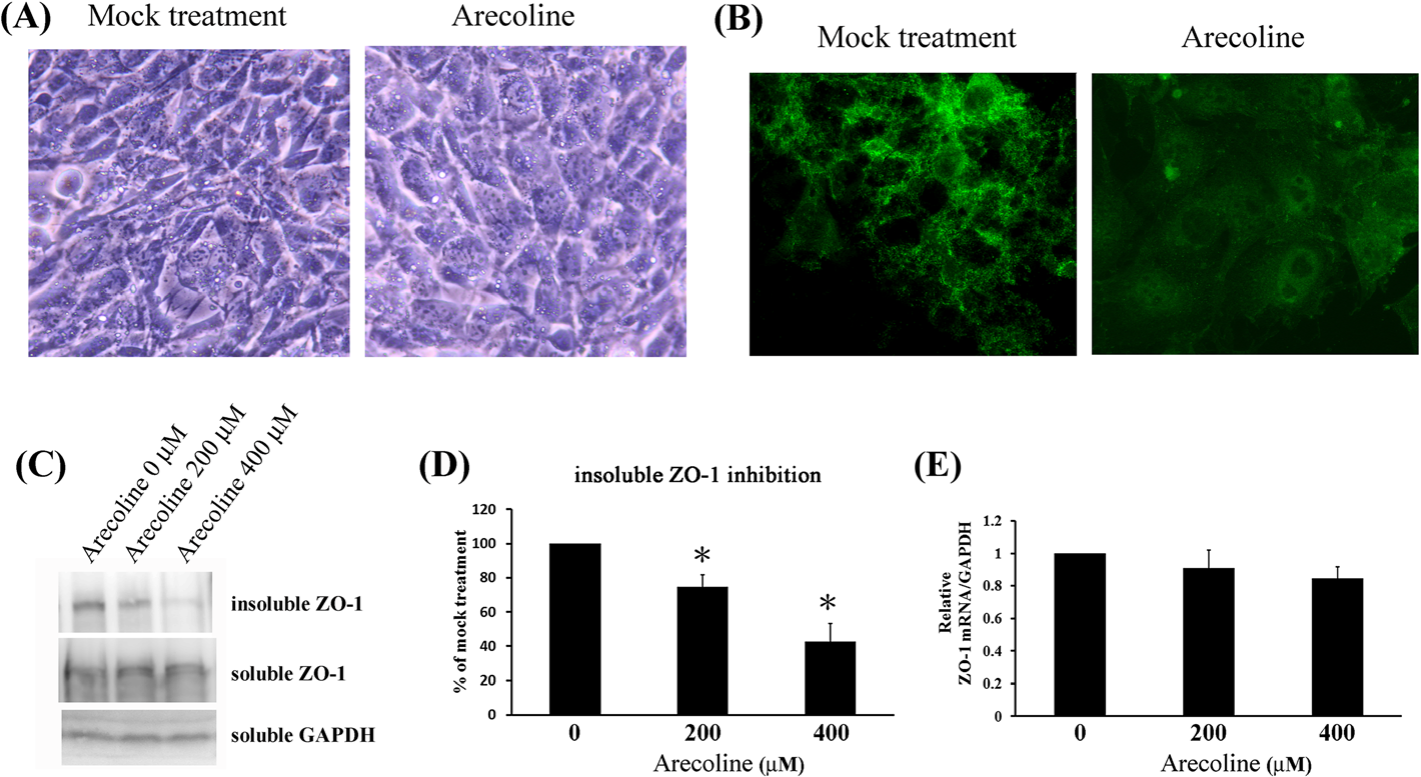
**Arecoline decreased membrane expression of ZO-1 in confluent TM4 cells.** These were treated with indicated arecoline concentration for 6 hours. **(A)** Morphology of confluent TM4 treated with (right panel) or without (left panel) 400 μM of arecoline were visualized. Treated cells (right panel) displayed flat and spread morphology compared to controls (left panel). **(B)** ZO-1 protein expression detected by immunofluorescent assay was visualized by fluorescent microscope. Arecoline-treated cells were manifested lower ZO-1 signal than controls. **(C)** Insoluble and soluble fractions of cell lysates were prepared for Z0-1 detection by Western blot. **(D)** Inhibitory effect of arecoline on insoluble ZO-1 is plotted on bar graph. Signals of insoluble ZO-1 were quantified by densitometry analysis and expressed as average percentage of respective control cells from three independent experiments to rate arecoline’s inhibitory effect (*P < 0.05 versus 0 μM controls). **(E)** The amounts of ZO-1 mRNA were determined using RT-qPCR. Results depict mean ± SD of three independent experiments. *P < 0.05 compared with cells incubated without arecoline treatment (0 μM).

Toxicants can induce intracellular junctional instability by decrease or redistribution of the junctional proteins between Sertoli and germ or Sertoli and Sertoli cells. ZO-1 is one of their target proteins. To test whether arecoline targets on ZO-1 in testis cells, a mouse Sertoli cell line TM4 is used. TM4 cells were treated with 400 μM of arecoline for 6 hours, direct effect of arecoline on cell morphology was confirmed by microscopic analysis. No any cell loss was observed after arecoline treatment (Figure 1A).

Though cells were more flat and spreaded than controls, arecoline did not change cell morphology, formation of bubble or death-like morphology. This result indicated that cytotoxicity might not be a direct effect via arecoline in confluent TM4 cells. To test ZO-1 inhibition via arecoline, ZO-1 proteins were imunostained and visualized by fluoresce microscope (Figure 1B). The punctate signal of ZO-1 on plasma membrane was stained in control cells, stronger signal detected between adjacent cells. Treated cells showed decreased expression of ZO-1 protein. Unlike crowded and closed cell morphology of controls, arecoline-treated cells showed flattened and loosened cell morphology. It has been reported that association of ZO-1 with Triton-insoluble fraction (fraction of membrane cytoskeleton) correlates with junctional integrity [30]. To evaluate effects of arecoline on junctional integrity, cells were treated with 400 μM of arecoline for 6 hours and ZO-1 protein in Triton-(in)soluble fractions was assessed by Western blot. While level of soluble ZO-1 rose, arecoline significantly reduced insoluble ZO-1 (Figure 1C). At arecoline concentration of 200 and 400 μM, levels of insoluble ZO-1 fell to 74.7 ± 7.2% and 42.5 ± 10.7%, as compared with mock control (Figure 1D). Effect of arecoline on ZO-1 mRNA was tested by qRT-PCR. Although level of ZO-1 mRNA in arecoline treated cells seemed to decreased, the results showed non-significant inhibition of ZO-1 mRNA by arecoline at 6 hours of treatment (Figure 1E). Taken together, these results indicate arecoline decreasing membrane expressions of ZO-1 protein in TM4 cells.

### Arecoline induces changes on phosphorylation of multiple signaling proteins

Our recent study indicated that ANE induced ROS-related oxidative stress in the testis [16]. Here, we found arecoline inhibited ZO-1 membrane expression, thus we speculated that arecoline may induce decreased junction integrity by a molecular mechanism similar to other toxicants.

To screen for signaling or proteins modulated by arecoline, Micro-Western Array [24,25], a recently developed high-throughput Western blotting analysis, was used (Additional file 2: Figure S1). TM4 cells were treated with 400 μM of arecoline, cell lysates isolated at 10 and 60 minutes for this analysis. We choice 48 antibodies capable of determining proteins involving in reproduction related signaling transduction (Additional file 1: Table S1). As shown in Table 1, arecoline increased the protein expression of phospho-p44/42 MAPKs(ERK1/2) Thr202/Tyr204, phospho-p38 MAPK Thr180/Tyr182, phospho-IkappaB-alpha Ser36, phospho-c-Jun Ser63 and phospho-mTOR Thr2446. It also decreased the protein abundance of phospho-PP2A Tyr307, phospho-STAT1 Ser727 and phospho-Src Tyr418.

**Table 1:** **Data collection of significant fold-changes for detected proteins from Micro-Western Array analysis**

		**Arecoline treated time**	
	**Antibodies**	**10 minutes**	**60 minutes**
Increased	Phospho-p44/42 MAPK (Thr202/Tyr204)	1.98	
	Phospho-p38 MAPK (Thr180/Tyr182)	1.51	
	Phospho-IkappaB-alpha (Ser36)		1.33
	Phospho-Jun(−c) (Ser63)		1.6
	Phospho-mTOR (Thr2446)	1.16	2.3
Decreased	Phospho-PP2A (Tyr307)	0.89	0.68
	Phospho-STAT1 (Ser727)		0.6
	Phospho-Src (Tyr418)	0.85	0.79

Note: Relative protein abundance was normalized to the average of GAPDH and actin. A fold change of 1.3 or 0.7 in expression between 0 and 10 minutes or 0 and 60 minutes groups was used as a cutoff.

**Figure 2:**
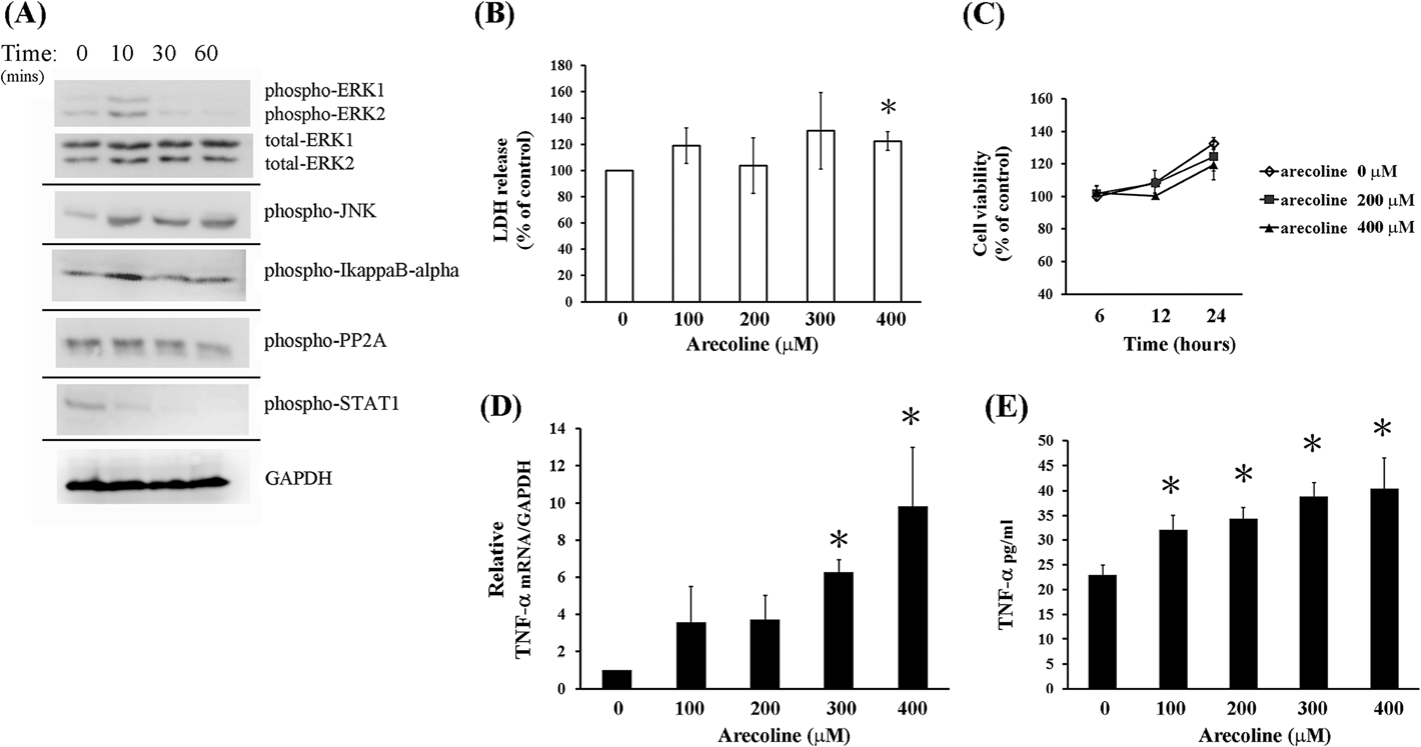
**Arecoline activates ERK1/2 MAPKs and increases TNF-alpha production. (A)** Protein signal of phospho-ERK1/2, ERK1/2, phospho-JNK, phospho-IkappaB-alpha, phospho-PP2A, phospho-STAT1 and GAPDH in arecoline (400 μM) treated cells were detected by Western blot. Arecoline induces phosphorylation of ERK1/2 MAPKs. These same membranes were stripped and re-detected by antibodies of total ERK1/2 and GAPDH. Results shown represent three independent experiments. **(B) (C)** No obvious cell death in arecoline-treated TM4 cells treated with indicated arecoline concentration, cytotoxicity or viability gauged by released LDH activity detection kit after 6 hours **(B)** or MTS Assay after 6, 12 and 24 hours of treatment **(C)**. Data express percentage of control cells (*P < 0.05 versus 0 μM control group). **(D) (E)** Arecoline induced TNF-alpha mRNA **(D)** and soluble protein production **(E)**. Cells were treated with indicated concentration of arecoline for 6 hours, levels of TNF-alpha mRNA measured by RT-qPCR and soluble protein in culture medium measured by ELISA. Results depict mean ± SD of three independent experiments. *P < 0.05 compared with cells incubated without arecoline treatment (0 μM).

We next confirmed the results of Micro-Western Array by traditional Western blotting analysis (Figure 2A). Arecoline induced phosphorylation of ERK1/2 MAPKs and IkappaB-alpha. It also decreased signal of phospho-PP2A and STAT1 in TM4 cell. The results of Micro-Western Array suggest that arecoline induced changes on phosphorylation of c-Jun, p38 MAPK, mTOR and Src. Changes in these phosphorylated signal were hardly determined perhaps due to inappropriate conditions or antibodies. It is well known that c-Jun is a downstream molecule of c-Jun N-terminal kinase (JNK) pathway [31]. We therefore alternately tried to rate the phosphorylation of JNK. Indeed, levels of phosphorylated JNK were increased at 10 minutes after arecoline stimulation in TM4 cells.

Arecoline is reported to induce cytotoxic effects in various cell lines, which is mentioned as a major cause of Betel quid chewing-related diseases. To confirm whether cytotoxiticity is a direct effect by arecoline on male reproductive dysfunction, cells were treated with different doses of arecoline for 6 hours and lactate dehydrogenase (LDH) assay performed (Figure 2B). After 6 hours of arecoline treatment, LDH activity was slightly increased, with significantly effect observed at concentration of 400 μM (122.6 ± 7%, *p* < 0.05) versus mock control. Differences between other arecoline-treated groups and mock control were insignificant, indicating that arecoline caused little damage to cells. We further tested the effects of arecoline on cell viability by MTS cell viability assay (Figure 2C). Arecoline treatment did not decrease cell viability in 6 hours and 12 hours. Slight reduction (about 10%) appeared at 24 hours of treatment, without statistical significance between control and arecoline treated groups. The results imply that cytotoxicity is not a direct effect on sperm abnormalities via arecoline.

### Arecoline induces TNF-alpha production

Toxicants increase proinflammatory cytokine (TNF-alpha) expression, which mediates disruption of BTB by down-regulation of cell junction proteins [32]. TNF-alpha and other proinflammatory cytokines have been reported to down-regulate or redistribute cell junction proteins (e.g. occludin, ZO-1 and N-cadherin), which leads to junction disruption of cells in testis. Hence, TNF-alpha and other proinflammatory cytokines are important mediator or stimulator in modulation of male reproductive functions [33]. It is reported that arecoline stimulation increases TNF-alpha production in leukemia culture cells [34]. To ascertain if arecoline stimulation increases TNF-alpha production, TM4 cells were treated with arecoline for 6 hours and then for expressions of TNF-alpha mRNA (Figure 2D) and secreted soluble protein (Figure 2E). Arecoline treatment, induced TNF-alpha mRNA and soluble protein expression in a dose-dependent manner, significant induction was observed at 300 μM or 100 μM concentration respectively. This result indicated that arecoline increased TNF-alpha production in TM4 cells.

### Arecoline induces TNF-alpha production and ZO-1 redistribution through ERK1/2 activation

ERK1/2 MAPKs signaling pathway has been reported to play critical role in male reproductive toxicity. Activation of ERK1/2 MAPKs stimulated by ANE or arecoline has also been reported previously [35,36]. Therefore, to test whether arecoline induced ERK1/2 pathway to regulate TNF-alpha and ZO-1, we used PD98059, ERK1/2 specific inhibitor.

**Figure 3:**
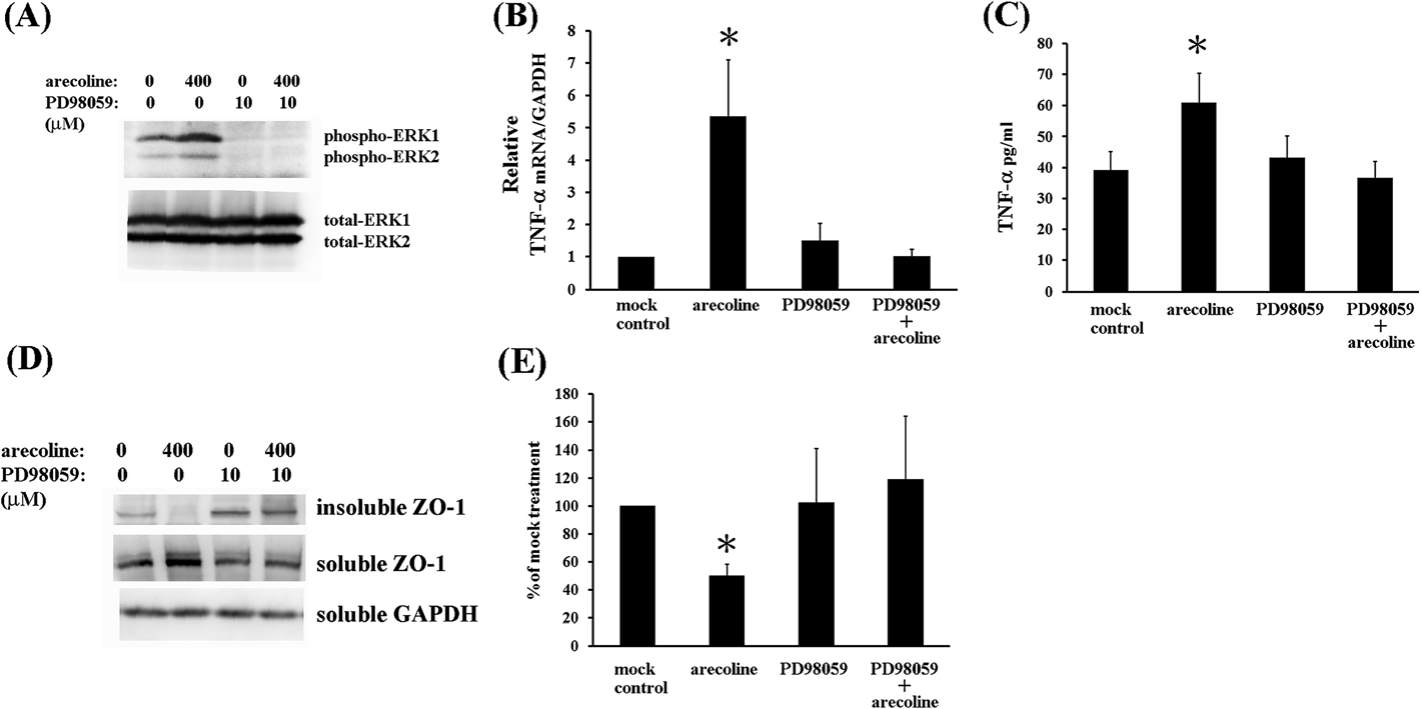
**Activation of ERK1/2 MAPKs is important for TNF-alpha induction and ZO-1 protein redistribution by arecoline in TM4 cells. (A)** PD98059 pretreatment inhibited ERK1/2 activation. Cells were stimulated with or without 400 μM of arecoline for 10 minutes after pretreatment with 10 μM PD98059 for 1 hour, levels of ERK1/2 and phospho-ERK1/2 determined by Western blot. Results shown represent three independent experiments. **(B) (C)** PD98059 pretreatment inhibited arecoline-induced increase in mRNA **(B)** and soluble protein **(C)** of TNF-alpha. Cells were treated with or without arecoline for 6 hours after PD98059 pretreatment. Levels of mRNA and soluble protein of TNF-alpha were measured by RT-qPCR and ELISA respectively. Results depict mean ± SD of three independent experiments. *P < 0.05 compared with cells incubated without arecoline treatment (mock control). **(D)** PD98059 pretreatment rescued arecoline-induced protein redistribution of ZO-1. Levels of insoluble or soluble ZO-1 were determinate by Western blot. Results represent three independent experiments. **(E)** Effect of arecoline and PD98059 on insoluble ZO-1 is plotted on bar graph. Signals were quantified by densitometry analysis and expressed as average percentage of respective control cells from three independent experiments (*P < 0.05 versus mock controls).

Confluent TM4 cells were pretreated with or without 10 μM of PD98059 for 1 hour and then incubated in the presence or absence of 400 μM arecoline for 10 minutes. Apparently, PD98059 inhibited the basal and arecoline increased ERK1/2 phosphorylation (Figure 3A). We next examined effect of PD98059 pretreatment on TNF-alpha production and ZO-1 protein expression in cells treated with arecoline for 6 hours. Level of mRNA and secreted soluble protein of TNF-alpha did not change significantly via PD98059 alone, as measured by RT-qPCR (Figure 3B) and ELISA analysis (Figure 3C). Furthermore, arecoline-treated cells showed increased TNF-alpha mRNA and soluble protein, whereas PD98059 treatment prevented arecoline effect on TNF-alpha expression.

In arecoline-treated cells, protein levels of insoluble ZO-1 were decreased to 50.2 ± 7.9%, as compared with mock control (Figure 3D and E). As results shown in Figure 1C, soluble ZO-1 was increased. PD98059 alone slightly increased insoluble ZO-1 expression without statistic significance. Importantly, PD98059 pretreatment rescued arecoline-induced changes on insoluble and soluble ZO-1, suggesting that ERK1/2 suppression could block the effect via arecoline on ZO-1 redistribution. These results prove arecoline induced TNF-alpha level and ZO-1 redistribution through ERK1/2 activation.

**Figure 4:**
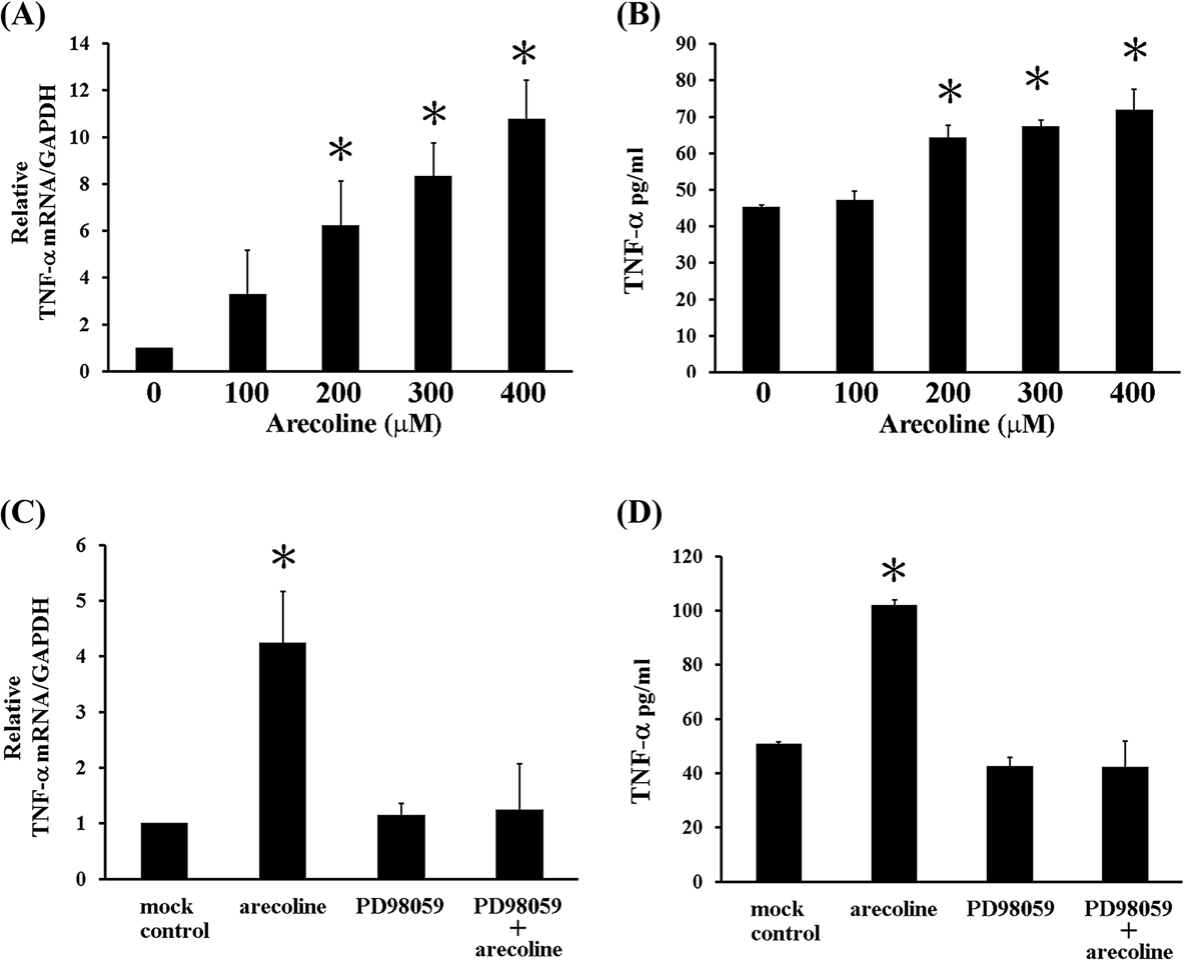
**ERK1/2 MAPKs are involved in increases of mRNA and soluble protein TNF-alpha by arecoline in THP1 cells. (A) (B)** Arecoline increased mRNA **(A)** and soluble protein **(B)** of TNF-alpha. THP1 (2 × 10^6^ in 1.5 mL) cells were treated with indicated doses of arecoline for 6 hours. **(C) (D)** PD98059 inhibited TNF-alpha mRNA **(C)** and soluble protein **(D)** inductioin via arecoline. Cells were pretreated with 10 μM PD98059 for 1 h and further treated with 400 μM arecoline for 6 hours. Those mRNA and soluble protein of TNF-alpha levels were measured by RT-qPCR and ELISA. These results represent mean ± SD of three independent experiments; asterisk shows significant difference (p < 0.05) compared with controls (0 μM).

Macrophages and monocytes are important sources of TNF-alpha [37]. Therefore, we further tested the effects of arecoline on TNF-alpha production using human monocytic cell line THP-1. After 6 hours of treatment, arecoline increased TNF-alpha mRNA expression (Figure 4A) and soluble protein expression (Figure 4B) in THP1 cells. Similarly, pretreatment with PD98059 reversed arecoline effect (Figure 4C and D). All these Results indicated that ERK1/2 activation may involved in TNF-alpha induction by arecoline in THP1 cells.

## Discussion

During betel chewing, arecoline can diffuse into blood through buccal mucosa or be absorbed by gastrointestinal tract [38]. The concentration of arecoline in saliva was reported to reach around 140 μg/ml (about 902.12 μM) [39]. Arecoline can be detected in blood, placenta [40], urine [41] or breast milk [42]. However, reports or information on the levels of arecoline or other alkaloids in testis are lacking. Although male reproductive toxicity via areca nut or arecoline has been observed in several *in vivo* studies [13,17], appropriate dose of alkaloids treatment for *in vitro* studies is a controversial issue. *In vitro* studies of arecoline effects on sperm motility or morphology by using dosages of 50 to 300 μg/ml (about 322 to 1933 μM) have been reported previously [14,15]. In this study, we used 100 to 400 μM of arecoline treatment for investigating its possible direct or accumulated effect on Sertoli cells.

Arecoline is cytotoxitic to various cells: e.g., normal human gingival fibroblasts (HGF) cell etc. [5]. Those are implicated in initial pathogenesis of areca nut-linked disease. Arecoline induced ROS production, and leads to cell cycle arrest, DNA damage and finally causes cell death. They are critical mediators of arecoline-induced cytotoxicity. In this present study, we found that, arecoline increased TNF-alpha and ZO-1 redistribution without inhibiting cell viability. Nevertheless, we did not ignore cytotoxic effects of arecoline because of short treatment time. Owing to our results, we speculate that induction of proinflammatory cytokines and decrease of junctional integrity are early events of arecoline effect on BTB damage before cytotoxicity.

In this study, we used Micro-Western Arrays to detect which signaling molecules activated after arecoline stimulation. Micro-Western Array, a newly developed assay for proteomic analysis, is modified from reverse phase protein array [43]. It is more precise than common dot blot or antibody array and suitable for detection of protein abundance or modification status. Studies on anti-cancer activity of triol and EGF receptor signaling employed this method [24,44]. The MAPKs signaling is not only a stress-responsive signaling, but it is also known to regulate multiple processes of male reproduction [45]. In maintenance of BTB junctional integrity, activation of ERK1/2 leads to restructure BTB, disrupting BTB [46] and germ cell adhesion [47,48]. Our results showed arecoline inducing activation of ERK1/2 MAPKs in TM4 cells; while expecting, this result is crucial. Numerous environmental toxicants cause male reproductive dysfunction through ERK1/2 MAPK mediating pathways [19,49]; arecoline may exert similar effects as other toxicants. Previous related studies indicate that exposure to environmental toxicants activates MAPKs (ERK and JNK/p38) and then induces proinflammatory cytokines (TGF-beta, TNF-alpha, or IL-6) via oxidative stress [50-52]. Cytokines and activated MAPKs together result in internalization of tight and adherens junction proteins mediated by protein redistribution and endocytic vesicle [20,53]. Besides this, cytokine induction also stimulates cells to activate MAPKs signaling, which further amplifies toxic effects. Those effects could be blocked by specific inhibitors of MAPKs, confirmed the regulated role of MAPKs activation [19,20].

Our results of Western blotting also indicated an increase on JNK phosphorylation via arecoline stimulation. Cadmium, a well known toxicant, has been shown to induce TGF-beta production and redistribution of tight junction proteins through p38 and JNK MAPKs pathways in rat testis or rat culture cells [23]. Inhibition of JNK signaling suppressed induction of connective tissue growth factor in buccal mucosal fibroblasts [54] or hemeoxygenase-1 in vein endothelial cells via arecoline [55]. Although our data indicated ERK1/2 inhibitor cause abolition of arecoline effects on TNF-alpha and ZO-1, we do not rule out the regulated role of JNK signaling in arecoline’s action. TNF-alpha secreted by sertoli and germ cells during spermatogenesis [52] or macrophages and monocytes stimulated by environmental toxicants has been proven to disrupt the BTB. TNF-alpha, a pleiotropic cytokine in testis, induces apoptosis of germ cells during spermatogenesis [18]. In addition to cause redistribution of junction proteins directly, changes in levels of testosterone were shown in TNF-alpha treated Leydig cells or TNF-alpha knock out mice [56,57]. Because testosterone is essential hormone for maintance of steroidogenesis, testosterone production might be involved in the in vivo effects of arecoline on male inferitility. Indeed, increased testotsterone levels and activity of related biosysthsis-enzyme has been shown in arecoline treated rats [58]. Abnormality on testosterone production might be another important pathway for arecoline’s effect.

ZO-1 is a well-known tight junction protein and important marker for integrating tight junction [59]. It usually binds to actin cytoplasmic filaments and another tight junction protein- occludin that are down-regulated in highly invasive cancer cells [60]. It is well known that dissociation of ZO-1 from the junctional complex is followed with increased permeability [61]. Our results indicated that arecoline decreases insoluble ZO-1 and increases soluble ZO-1, which represents redistribution of ZO-1 caused by arecoline. Since ZO-1 functions with other membrane junctional proteins, reduction of insoluble ZO-1 also represents decreased binding of ZO-1 to cytoskeleton, adherens junctions, or polarity complexes. Association of ZO-1 with Triton-insoluble fraction (fraction of membrane cytoskeleton) correlating with junctional integrity has been denomostrated previously [30]. Otherwise, our preliminary results also indicated inhibitory effect on membrane expression of occludin via arecoline in TM4 cells (Additional file 3: Figure S2A and B). Nonetheless, arecoline might inhibit occludin through other pathways since PD98059 could not stabled rescue occludin inhibition via arecoline (Additional file 3: Figure S2C and D). Although reduced expression and disrupted distribution of ZO-1 in arecoline-mediated oral carcinogenesis has been cited by Giri et al.’s, two different observations were shown in our study [62]. Firstly, our data indicated protein redistribution of ZO-1 was the primarily effect of arecoline. Second, we found that arecoline’s effect was through ERK1/2 activation. Importantly, we found arecoline inducing cellular protein redistribution of ZO-1 in TM4 sertoli cells, which was an important clue in molecular mechanisms of male reproductive dysfunction via areca nut. Arecoline reportedly can cross the blood brain barrier (BBB) [63]. The BTB, like BBB, also serves as an immunological barrier to protect meiotic division and post-meiotic germ cell development from threats via systemic circulation [18]. This study showed that arecoline induced ERK1/2 signaling, TNF-alpha production, and suppression of ZO-1 membrane expression, which correspond to mechanisms of BTB disruption via environmental toxins or drugs. Cytotoxicity of arecoline might amplify disruptive effect on BTB. Damage to BTB triggers loss of germ cell and decreased sperm quality or quantity, thereby causing male reproductive dysfunction.

## Conclusions

In conclusion, our study demonstrated ZO-1 redistribution and TNF-alpha induction by arecoline in TM4 cell. We also observed arecoline increased of TNF-alpha in THP1 cells. ERK1/2 MAPKs participates in those effects. Results imply reduced junctional integrity as early events of reproductive inhibition via arecoline before cytotoxicity.

## Additional files

Additional file 1: Table S1. List of antibodies used for Micro-Western Array analysis. Briefly information of used antibodies was shown. For data calculation, protein signals from actin and GAPDH were used as loading controls. Additional file 2: Figure S1. Analysis of protein expression in TM4 cells treated with arecoline by Micro-Western Array assay. Confluent cells (indicated as minute 0) were treated with 400 μM of arecoline for 10 or 60 minutes, and cell lysates were collected as previously described [23]. Changes in abundance of indicated proteins or their phosphorylated forms were determined by Micro-Western Array. Here, used 48 antibodies are listed in Additional file 1: Table S1. As shown in this figure, right half of blot (well A7-H12) was the duplicate of the left half (well A1-H6). Six samples printed in each well (from left to right) were cells treated with arecoline for 0, 10, 60 minutes (1-3) and condition controls (4-6), respectively. Artificial coloring differentiates the used secondary antibodies in species (red and green for anti-rabbit and anti-mouse, respectively). Selected data of relative protein abundance are listed in Table 1. Additional file 3: Figure S2. ERK1/2 inhibitor did not fully rescue protein redistribution of occludin via arecoline in TM4 cells. Treated methods are same as Figure 3. (A) (C) Detection of occludin in insoluble and soluble fractions of cell lysates by Western blot. Results represent three independent experiments. (B) (D) Effect of arecoline or PD98059 on insoluble occludin is plotted on bar graph. Insoluble occludin signals were quantified by densitometry analysis and expressed as average percentage of respective control cells from three independent experiments to rate arecoline’s effect (*P<0.05 versus 0 μM controls).
