# Supplementary material for: Arecoline induces TNF-alpha production and Zonula Occludens-1 redistribution in mouse Sertoli TM4 cells
Source: J Biomed Sci. 2014 Sep 9;21(1):93. doi: 10.1186/s12929-014-0093-z (PMC4256803; doi:10.1186/s12929-014-0093-z)
Supplement: Additional file 1: Table S1. — List of antibodies used for Micro-Western Array analysis. Briefly information of used antibodies was shown. For data calculation, protein signals from actin and GAPDH were used as loading controls. [file 12929_2014_93_MOESM1_ESM.docx]

**Supplementary tables**

Table S1: List of antibodies used for Micro-Western Array analysis

| NO. | Mico-Western position | Antibodies | Host species | Antibodies producers |
| --- | --- | --- | --- | --- |
| 1 | A1 | HSP70 | Rabbit | Cell Signaling Technology, Danvers, MA |
| 2 | A2 | Androgen Receptor clone PG-21 | Rabbit | Millipore Corp., Billerica, MA |
| 3 | A3 | Phospho-CREB (Ser133) clone 87G3 | Rabbit | Abcam, Cambridge, UK |
| 4 | A4 | PLC y-1 | Rabbit | OriGene Technologies, Inc., Rockville, MD |
| 5 | A5 | Phospho-IGF-IR (Tyr1131)/InsR (Tyr1158) clone JY202 | Mouse | Millipore Corp., Billerica, MA |
| 6 | A6 | Phospho-mTOR (Thr2446) | Rabbit | Millipore Corp., Billerica, MA |
| 7 | B1 | EGF Receptor clone D38B1 | Rabbit | Cell Signaling Technology, Danvers, MA |
| 8 | B2 | Actin | Rabbit | Novus Biologicals, Littleton, CO |
| 9 | B3 | Phospho-Kit(-c) (Tyr719) Antibody | Rabbit | Cell Signaling Technology, Danvers, MA |
| 10 | B4 | Phospho-PKR (Thr451) | Rabbit | Millipore Corp., Billerica, MA |
| 11 | B5 | Phospho-PP2A (pY307) RabMAb^®^ | Rabbit | Abcam, Cambridge, UK |
| 12 | B6 | Phospho-Insulin Receptor (Tyr 1150/1151) clone 10C3 | Mouse | Millipore Corp., Billerica, MA |
| 13 | C1 | Apolipoprotein E | Goat | Millipore Corp., Billerica, MA |
| 14 | C2 | E-Cadherin RabMAb^®^ | Rabbit | Abcam, Cambridge, UK |
| 15 | C3 | Phospho-p44/42 MAPK (Erk1/2) (Thr202/Tyr204) clone D13.14.4E | Rabbit | Cell Signaling Technology, Danvers, MA |
| 16 | C4 | Phospho-mTOR (Ser2481) Antibody | Rabbit | Cell Signaling Technology, Danvers, MA |
| 17 | C5 | Phospho-Akt1/PKBα (Ser473) clone 11E6 | Mouse | Millipore Corp., Billerica, MA |
| 18 | C6 | Phospho-Jun(-c) (Ser63) clone Y172 | Rabbit | Millipore Corp., Billerica, MA |
| 19 | D1 | PIAS1 RabMAb^®^ | Rabbit | Abcam, Cambridge, UK |
| 20 | D2 | Cyclin D1 clone EP272Y | Rabbit | Millipore Corp., Billerica, MA |
| 21 | D3 | Phospho-Met (Tyr1003) clone 13D11 | Rabbit | Cell Signaling Technology, Danvers, MA |
| 22 | D4 | Phospho-SAPK/JNK (Thr183/Tyr185) clone 81E11 | Rabbit | Millipore Corp., Billerica, MA |
| 23 | D5 | Phospho-Abl (Tyr245) | Rabbit | Millipore Corp., Billerica, MA |
| 24 | D6 | Phospho-Myc(-c) (Thr58/Ser62) | Rabbit | Millipore Corp., Billerica, MA |
| 25 | E1 | PP2A clone PPP2CA | Rabbit | Abcam, Cambridge, UK |
| 26 | E2 | Estrogen Receptor α clone E115 | Rabbit | Millipore Corp., Billerica, MA |
| 27 | E3 | Phospho-Tuberin/TSC2 (Thr1462) clone 5B12 | Rabbit | Cell Signaling Technology, Danvers, MA |
| 28 | E4 | Phospho-Met (Tyr1234/1235) clone D26 | Rabbit | Cell Signaling Technology, Danvers, MA |
| 29 | E5 | Phospho-Insulin Receptor (Tyr 1322) clone 21G12 | Mouse | Millipore Corp., Billerica, MA |
| 30 | E6 | Phospho-p38 MAPK (Thr180/Tyr182) clone D3F9 | Rabbit | Cell Signaling Technology, Danvers, MA |
| 31 | F1 | PIAS2 RabMAb^®^ | Rabbit | Abcam, Cambridge, UK |
| 32 | F2 | nNOS/NOS I | Rabbit | Millipore Corp., Billerica, MA |
| 33 | F3 | Phospho-Src (Tyr527) Antibody | Rabbit | Cell Signaling Technology, Danvers, MA |
| 34 | F4 | Phospho-IkappaB-alpha (pS36) RabMAb^®^ | Rabbit | Abcam, Cambridge, UK |
| 35 | F5 | Phospho-Src (Tyr416) clone 9A6 | Mouse | Millipore Corp., Billerica, MA |
| 36 | F6 | Phospho-Abl (Tyr412) | Rabbit | Millipore Corp., Billerica, MA |
| 37 | G1 | STUB1 RabMAb^®^ | Rabbit | Abcam, Cambridge, UK |
| 38 | G2 | Peroxisome Proliferator Activated Receptor | Mouse | Millipore Corp., Billerica, MA |
| 39 | G3 | Phospho-VEGF Receptor 2 (Tyr1059) clone D5A6 | Rabbit | Cell Signaling Technology, Danvers, MA |
| 40 | G4 | Phospho-PKCα (Thr638) | Rabbit | Millipore Corp., Billerica, MA |
| 41 | G5 | Phospho-EGFR (Tyr1173) clone 9H2 | Mouse | Millipore Corp., Billerica, MA |
| 42 | G6 | Phospho-Src (Tyr418) | Rabbit | Millipore Corp., Billerica, MA |
| 43 | H1 | PPARγ | Rabbit | Millipore Corp., Billerica, MA |
| 44 | H2 | GAPDH | Rabbit | Abcam, Cambridge, UK |
| 45 | H3 | Phospho-Akt (Thr308) | Rabbit | Cell Signaling Technology, Danvers, MA |
| 46 | H4 | Phospho-JNK (Thr183/Tyr185, Thr221/Tyr223) | Rabbit | Millipore Corp., Billerica, MA |
| 47 | H5 | Phospho-STAT1 (Ser727) | Rabbit | Millipore Corp., Billerica, MA |
| 48 | H6 | Phospho-FAK (Tyr861) | Rabbit | Millipore Corp., Billerica, MA |
