# Supplementary figures and images for: Arecoline induces TNF-alpha production and Zonula Occludens-1 redistribution in mouse Sertoli TM4 cells
Source: J Biomed Sci. 2014 Sep 9;21(1):93. doi: 10.1186/s12929-014-0093-z (PMC4256803; doi:10.1186/s12929-014-0093-z)

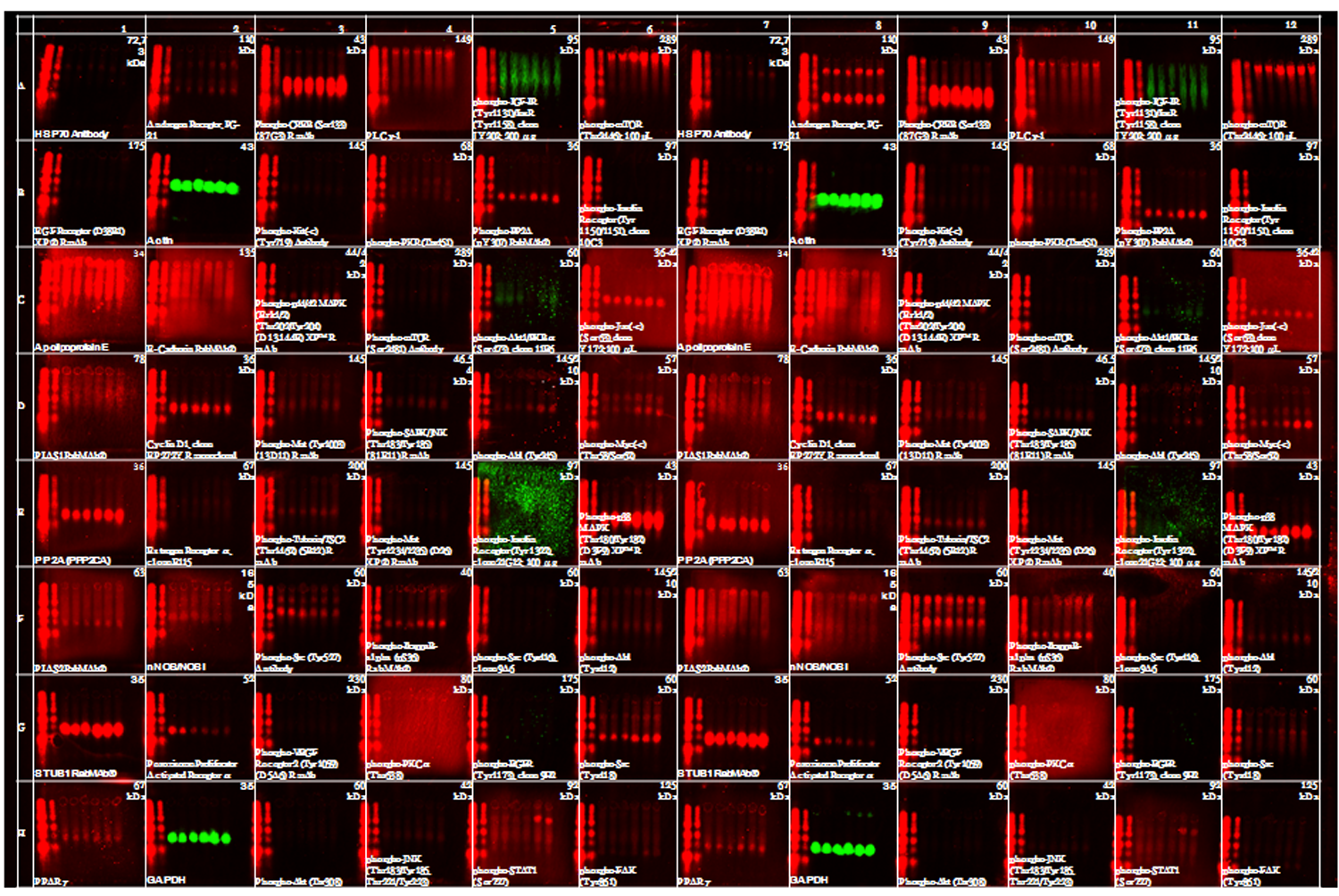

Supplement: Additional file 2: Figure S1. — Analysis of protein expression in TM4 cells treated with arecoline by Micro-Western Array assay. Confluent cells (indicated as minute 0) were treated with 400 μM of arecoline for 10 or 60 minutes, and cell lysates were collected as previously described [23]. Changes in abundance of indicated proteins or their phosphorylated forms were determined by Micro-Western Array. Here, used 48 antibodies are listed in Additional file 1: Table S1. As shown in this figure, right half of blot (well A7-H12) was the duplicate of the left half (well A1-H6). Six samples printed in each well (from left to right) were cells treated with arecoline for 0, 10, 60 minutes (1-3) and condition controls (4-6), respectively. Artificial coloring differentiates the used secondary antibodies in species (red and green for anti-rabbit and anti-mouse, respectively). Selected data of relative protein abundance are listed in Table 1. [file 12929_2014_93_MOESM2_ESM.tiff]

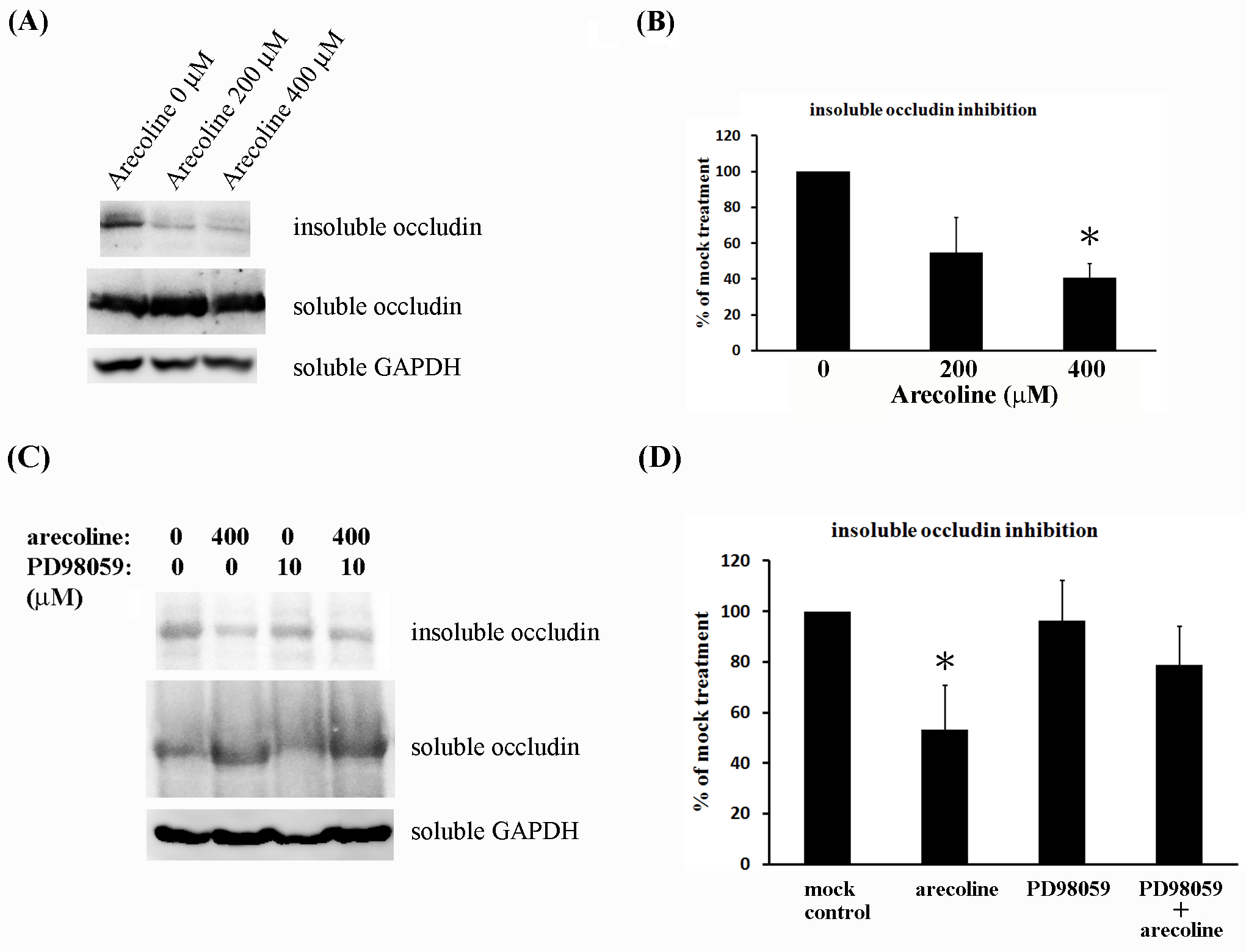

Supplement: Additional file 3: Figure S2. — ERK1/2 inhibitor did not fully rescue protein redistribution of occludin via arecoline in TM4 cells. Treated methods are same as Figure 3. (A) (C) Detection of occludin in insoluble and soluble fractions of cell lysates by Western blot. Results represent three independent experiments. (B) (D) Effect of arecoline or PD98059 on insoluble occludin is plotted on bar graph. Insoluble occludin signals were quantified by densitometry analysis and expressed as average percentage of respective control cells from three independent experiments to rate arecoline’s effect (*P<0.05 versus 0 μM controls). [file 12929_2014_93_MOESM3_ESM.tiff]
